# Exploring the use of body worn cameras in acute mental health wards: a mixed-method evaluation of a pilot intervention

**DOI:** 10.1186/s12913-024-11085-x

**Published:** 2024-05-29

**Authors:** Una Foye, Keiran Wilson, Jessica Jepps, James Blease, Ellen Thomas, Leroy McAnuff, Sharon McKenzie, Katherine Barrett, Lilli Underwood, Geoff Brennan, Alan Simpson

**Affiliations:** 1 Mental Health Nursing, Health Service and Population Research Department, Institute of Psychiatry, Psychology & Neuroscience, King’s College London, Denmark Hill, London, SE5 8AF UK; 2 Florence Nightingale Faculty of Nursing, Midwifery & Palliative Care, Mental Health Nursing, King’s College London, London, UK; 3 Lived Experience Advisor, London, UK

**Keywords:** Implementation, Body worn cameras, Staff, Qualitative, Mental health, Inpatient, Nursing

## Abstract

**Background:**

Body worn cameras (BWC) are mobile audio and video capture devices that can be secured to clothing allowing the wearer to record some of what they see and hear. This technology is being introduced in a range of healthcare settings as part of larger violence reduction strategies aimed at reducing incidents of aggression and violence on inpatient wards, however limited evidence exists to understand if this technology achieves such goals.

**Aim:**

This study aimed to evaluate the implementation of BWCs on two inpatient mental health wards, including the impact on incidents, the acceptability to staff and patients, the sustainability of the resource use and ability to manage the use of BWCs on these wards.

**Methods:**

The study used a mixed-methods design comparing quantitative measures including ward activity and routinely collected incident data at three time-points before during and after the pilot implementation of BWCs on one acute ward and one psychiatric intensive care unit, alongside pre and post pilot qualitative interviews with patients and staff, analysed using a framework based on the Consolidated Framework for Implementation Research.

**Results:**

Results showed no clear relationship between the use of BWCs and rates or severity of incidents on either ward, with limited impact of using BWCs on levels of incidents. Qualitative findings noted mixed perceptions about the use of BWCs and highlighted the complexity of implementing such technology as a violence reduction method within a busy healthcare setting Furthermore, the qualitative data collected during this pilot period highlighted the potential systemic and contextual factors such as low staffing that may impact on the incident data presented.

**Conclusion:**

This study sheds light on the complexities of using such BWCs as a tool for ‘maximising safety’ on mental health settings. The findings suggest that BWCs have a limited impact on levels of incidents on wards, something that is likely to be largely influenced by the process of implementation as well as a range of contextual factors. As a result, it is likely that while BWCs may see successes in one hospital site this is not guaranteed for another site as such factors will have a considerable impact on efficacy, acceptability, and feasibility.

**Supplementary Information:**

The online version contains supplementary material available at 10.1186/s12913-024-11085-x.

## Background

Body worn cameras (BWC) are mobile audio and video capture devices that can be secured to clothing allowing the wearer to record some of what they see and hear. In England, these have been introduced in the National Health Service (NHS) as part of a violence reduction strategy [1] which emphasises the reduction of aggression and violence against staff. The NHS Staff Survey 2022 found that 14.7% of NHS staff had experienced at least one incident of physical violence from patients, relatives or other members of the public in the previous 12 months. Violent attacks on staff were found to contribute to almost half of staff illness [2]. Levels of violence against staff working in mental health trusts remain much higher than other types of healthcare providers [3]. Numerous reports internationally highlight the increased risks faced by staff working in psychiatric care [4], though studies have reported that both ward staff and mental health patients experience violence and feeling unsafe on inpatient wards [5, 6].

Body worn cameras have been in use for over a decade within law enforcement, where they hoped to provide transparency and accountability within use-of-force incidents and in the event of citizen complaints against police [7]. It was believed that video surveillance would help identify integral problems within the organisation, improve documentation of evidence, reduce use-of-force incidents, improve police-community relations, and provide training opportunities for officers [8]. However, a recent extensive international systematic review by Lum et al. [9], found that despite the successes noted in early evaluations, the way BWCs are currently used by police may not substantially affect most officer or citizen behaviours. Irrespective of these findings, other public services such as train operators have been implementing BWCs for security purposes, with reductions reported in the number of assaults on railway staff [10].

A recent systematic review of BWC use in public sector services established that there is a poor evidence base supporting the use of BWCs in the reduction of violence and aggression [11]. Yet, we are seeing a swift increase in the use of BWCs in mental health settings with that aim, with few studies conducted on the use of BWC technology in inpatient mental health wards, and even fewer studies exploring staff or patients’ views. Two evaluations conducted in England reported mixed results with both increases and decreases in violence and aggression found, and variation between types of wards. There is some suggestion of a reduction in more serious incidents and the use of restraint, but quality of evidence is low [12, 13].

The use of BWCs in mental healthcare settings for safety and security remains a contentious topic due to the lack of evidence regarding the influence that such technology has on preventing violence and aggression and the complex philosophical and ethical issues raised, particularly where many patients may lack capacity and/or are detained under mental health legislation [14]. Additionally, there are concerns that BWCs may be used as a ‘quick fix’ for staff shortages rather than addressing the wider systemic and resourcing issues facing services [15]. With little independent evaluation of body-worn cameras in mental health settings, many of these concerns remain unanswered. There is also limited understanding of this technology from an implementation perspective. Therefore, in this study we aimed to conduct an independent evaluation of the introduction of BWCs as a violence reduction intervention on two inpatient mental health wards during a six-month pilot period to explore the impact of using the technology, alongside an exploration of the facilitators and barriers to implementation.

### Research aim(s)

To evaluate the implementation of BWCs on two inpatient mental health wards, including the impact on incidents, the acceptability to staff and patients, the sustainability of the resource use and ability to manage the use of BWCs on these wards.

## Methods

### Patient and public involvement

The research team included a researcher and independent consultant, each with lived experience of mental health inpatient care. In addition, we recruited and facilitated a six member Lived Experience Advisory Panel (LEAP). This group was made up of patients and carers, some of whom had experienced the use of BWCs. Members were of diverse ethnic backgrounds and included four women and two men. The LEAP provided guidance and support for the research team in developing an understanding of the various potential impacts of the use of BWCs on inpatient mental health wards. Members contributed to the design of the study, development of the interview schedule, practice interviews prior to data collection on the wards, and supported the analysis and interpretation of the data, taking part in coding sessions to identify themes in the interview transcripts. The LEAP met once a month for two hours and was chaired by the Lived Experience Research Assistant and Lived Experience Consultant. Participants in the LEAP were provided with training and paid for their time.

### Setting

The pilot introduction of the body worn cameras was conducted within a London mental health Trust consisting of four hospital sites with 17 acute wards. The research team were made aware of extensive preparatory work and planning that was conducted at a directorate and senior management level prior to camera implementation, including lived experience involvement and consultation, and the development of relevant policies and protocols inclusive of a human rights assessment and legal consultation.

The pilot period ran from 25th April to 25th October 2022. Reveal (a company who supply BWCs nationally across the UK) provided the Trust with 12 Calla BWCs for a flat fee that covered use of the cameras, cloud-based storage of footage, management software, and any support/maintenance required during the pilot period. Cameras were introduced to two wards based on two hospital sites, with six cameras provided to each of the wards on the same date. Training on using the BWCs was provided by the BWC company to staff working on both wards prior to starting the pilot period. Ward one was a 20-bed male acute inpatient ward, representing the most common ward setting where cameras have been introduced. Ward two was a ten-bed male Psychiatric Intensive Care Unit (PICU), representing smaller and more secure wards in which patients are likely to present as more unwell and where there are higher staff to patient ratios.

### Design

To answer our research questions, we used a mixed-methods design [16]. Using this design allowed us to investigate the impact of implementing BWCs in mental health settings on a range of quantitative and qualitative outcomes. This mixed methods design allows the study to statistically evaluate the effectiveness of using BWCs in these settings on key dependent variables (i.e., rates of violence and aggression, and incidents of conflict and containment) alongside qualitatively exploring the impact that the implementation of such technology has on patients and staff.

To ensure that the study was able to capture the impact and effect of implementation of the cameras, a repeated measures design was utilised to capture data at three phases on these wards:

1. Pre-pilot data: data prior of the implementation of the BWCs (quantitative and qualitative data).
2. Pilot period data: data collected during the six-month pilot period when BWCs were implemented on the wards (quantitative and qualitative data).
3. Post-pilot: data collected after the pilot period ended and cameras had been removed from the wards (quantitative data only).

### Quantitative methods

Quantitative data was collected at all three data collection periods:

1. Pre-period: Data spanning six months prior to the implementation of BWCs (Nov 21 to May 22).
2. Pilot period: Data spanning the six months of the Trusts pilot period of using BWCs on the wards (June 22 to Nov 22).
3. Post-pilot: Data spanning the six months following the pilot period, when BWCs had been removed (Dec 22 to May 23).

### Quantitative measures

To analyse the impact of BWC implementation, we collected two types of incident data related to violence and aggression and use of containment measures, including BWCs. Combined, these data provide a view of a wide range of incidents and events happening across the wards prior to, during, and after the implementation and removal of the BWCs.

#### The patient-staff conflict checklist

The Patient-staff Conflict Checklist (PCC-SR) [17] is an end of shift report that is completed by nurses to collate the frequency of conflict and containment events. This measure has been used successfully in several studies on inpatient wards [18–20].The checklist consists of 21 conflict behaviour items, including physical and verbal aggression, general rule breaking (e.g., smoking, refusing to attend to personal hygiene), eight containment measures (e.g., special observation, seclusion, physical restraint, time out), and staffing levels. In tests based on use with case note material, the PCC-SR has demonstrated an interrater reliability of 0.69 [21] and has shown a significant association with rates of officially reported incidents [22].

The checklist was revised for this study to include questions related to the use of BWCs (*e.g., how many uses of BWCs happened during the shift when a warning was given and the BWC was not used; when a warning was given and the BWC was used; when the BWC was switched on with no warning given*) in order to provide insight into how the cameras were being used on each ward (see appendix 1). Ward staff were asked to complete the checklist online at the end of each shift.

### Routinely collected incident data (via datix system)

To supplement the PCC-SR-R, we also used routinely collected incident data from both wards for all three data collection phases. This data is gathered as part of routine practice by ward staff members via the Datix system Datix [23] is a risk management system used widely across mental health wards and Trusts in the UK to gather information on processes and errors. Previous studies have utilised routinely collect data via this system [24, 25]. Incidents recorded in various Datix categories were included in this study (see Table 1). Incidents were anonymised before being provided to the research team to ensure confidentiality.

**Table 1:** Datix categories and aggregate variables

Aggregate variable	Datix labels
Violence (against person)	
	Actual Inappropriate Sexual Behaviour
	Actual Physical Assault By Patient On OTHER
	Actual Physical Assault By Patient On PATIENT
	Actual Physical Assault By Patient On STAFF
	Actual Sexual Assault By Patient On STAFF
	Aggression Resulting In Accidental Injury
	Alleged Sexual Assault By Patient On PATIENT
	Sexual Harassment By Patient On STAFF
Violence (against object)	
	Arson
	Patient - Hit Something Fixed/Stationary
	Patient - Suspected Arson
	Throwing Objects Aggressively
Verbal aggression	
	Alleged Harassment By Patient On OTHER
	Alleged Harassment By Patient On PATIENT
	Other Harassment By Patient On PATIENT
	Threatened Assault By Patient On OTHER (No Harm)
	Threatened Assault By Patient On STAFF (No Harm)
	Verbal Assault
Self-harm	

Routinely collected data included:

- Recorded incidents of violence and aggression.
- Recorded use of restrictive practices including seclusion, restraint, and intra-muscular medication/rapid tranquilisations.
- Patient numbers.
- Staffing levels.
- Numbers of staff attending BWC training.

### Quantitative data analysis

#### Incident reports

Incident reports retrieved from Datix were binary coded into aggregate variables to examine violence and aggression, self-harm, and other conflict as outlined in Table 1. Multivariate analyses of variance (MANOVA) were used to identify differences in type of incident (violence against person, violence against object, verbal aggression, self-harm, conflict) for each ward. MANOVA was also used to examine differences in incident outcomes (severity, use of restrictive practice, police involvement) across pre-trial, trial, and post-trial periods for each ward. Incident severity was scored by ward staff on a four-point scale (1 = No adverse outcome, 2 = Low severity, 3 = Moderate severity, 4 = Severe). Use of restrictive practice and police involvement were binary coded for presence or absence. Analyses were conducted using SPSS [26].

#### Patient-staff conflict checklist shift-report – revised (PCC-SR-R; )

Data were condensed into weeks for analysis rather than shifts to account for variability in PCC-SR-R submission by shift. Linear regressions assessed the relationship between BWC use and incident outcome (severity, use of restrictive practice, police involvement).

### Qualitative methods

We used semi-structured qualitative interviews to explore participants’ experiences of BWCs on the ward to understand the impact of their use as well as to identify any salient issues for patients, staff and visitors that align with the measures utilised within the quantitative aspect of this study. These interviews were conducted at two time points: pre-pilot and at the end of the six-month pilot period.

#### Sample selection, eligibility, and recruitment

Convenience sampling was used to recruit staff and patients on wards. Researchers approached ward managers to distribute information sheets to staff, who shared that information with patients. Staff self-selected to participate in the study by liaising directly with the research team. Patients that were identified as close to discharge and having capacity to consent were approached by a clinical member of the team who was briefed on the study inclusion criteria (see Table 2). The staff member spoke with the patient about the study and provided them with a copy of the information sheet to consider. If patients consented, a member of the research team approached the participant to provide more information on the study and answer questions. After initial contact with the research team, participants were given a 24-hour period to consider whether they wanted to participate before being invited for an interview.

**Table 2:** Inclusion and exclusion criteria

	Patient Eligibility	Staff Eligibility
**Inclusion Criteria**	• Patients currently on the selected mental health inpatient wards for adults of a working age (18+). • Capacity to give informed consent and participate in a research study. • Basic understanding of English (written or spoken).	• Staff currently working on or managing the selected mental health inpatient wards for adults of a working age (18+). • Capacity to give informed consent and participate in a research study. • Basic understanding on English (written or spoken).
**Exclusion Criteria**	• Patients lacking capacity to consent to take part in the research. • Patients who have not been on the selected mental health inpatient wards for adults of a working age (18+). • Children and adolescents under 18.	• Staff working on any other wards. • Unable to understand English.

### Procedure

Participants were invited to take part in an interview within a private space on the ward. Interviews were scheduled for one hour with an additional 15 min before and after to obtain informed consent and answer any questions. Participation was voluntary and participants were free to withdraw at any time. To thank patients for their time, we offered a £10 voucher following the interview. Interviews were audio-recorded and saved to an encrypted server. Interview recordings were transcribed by an external company, and the research team checked the transcripts for accuracy and pseudonymised all participants. All transcripts were allocated a unique ID number and imported to MicroSoft Excel [27] for analysis.

### Qualitative data analysis

Qualitative data were analysed using a framework analysis [28] informed by implementation science frameworks. Our coding framework used the Consolidated Framework for Implementation Research (CFIR) [29], which is comprised of five major domains including: Intervention Characteristics, Implementation Processes, Outer Setting, Inner Setting, and Characteristics of the Individual. Each domain consists of several constructs that reflect the evidence base of the types of factors that are most likely to influence implementation of interventions. The CFIR is frequently used to design and conduct implementation evaluations and is commonly used for complex health care delivery interventions to understand barriers and facilitators to implementation. Based on its description, the CFIR is an effective model to address our research question, particularly given the complexity of the implementation of surveillance technology such as BWCs in this acute care setting.

The initial analytic stage was undertaken by eight members of the study team with each researcher charting data summaries onto the framework for each of the interviews they had conducted on MicroSoft Excel [27]. Sub-themes within each broad deductive theme from our initial framework were then derived inductively through further coding and collaborative discussion within the research team, inclusive of Lived Experience Researcher colleagues. Pseudonyms were assigned to each participant during the anonymisation of transcripts along with key identifiers to provide context for illustrative quotes (e.g., P = patient, S = staff, A = acute ward, I = Intensive Care, Pre = pre-BWC implementation interview, Post = Post BWC implementation interview).

### Ethics

All participants gave their informed consent for inclusion before they participated in the study. The study was conducted in accordance with the Declaration of Helsinki, and the protocol was approved by the Health Research Authority: London - Camden & Kings Cross Research Ethics Committee (IRAS Project ID 322,268, REC Reference 23/LO/0337).

## Results

### Quantitative results

#### Exploring how body worn cameras were used during the pilot period

**Fig. 1:**
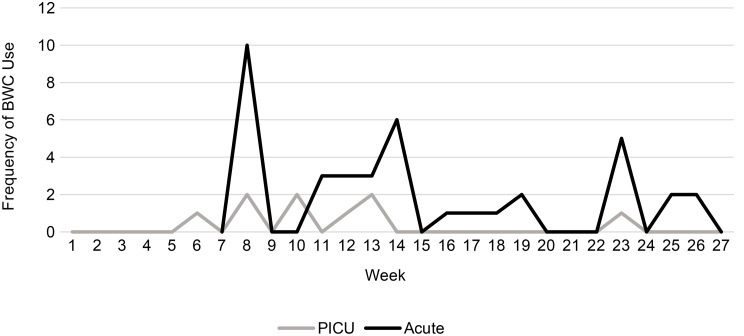
BWC use by ward per week of pilot (no data available before week 6 on Ward 1)

Analysis of the PCC-SR-R provides information about how the BWCs were used on a day-to-day basis during the pilot period. Out of 543 total shift reports completed, BWC use was reported 50 times, indicating that BWCs were used on less than 10% of shifts overall; 78% of those deployments were on the Acute ward (see Figure 1). Overall, the majority of deployments happened as activations without a warning being given (*n* = 30, 60% of activations), 19 times the BWC was deployed with a warning but the camera was not activated (38%), and only one was the camera activated without a warning being given (2%).

According to the PCC-SR-R, a total of 227 incidents of aggression occurred during the pilot period across both wards (see Table 3). Overall, there were small statistically significant correlations between BWC usage and certain types of conflict, aggression, and restrictive practice. Results found that BWC use was positively correlated with verbal aggression and use of physical restraint. BWC use was moderately positively correlated with verbal aggression (*r* = .37, *p* < .001). This indicates that BWCs were more likely to be used in incidents involving verbal aggression, which do not tend to be documented in Datix. Similarly, BWC use was moderately positively correlated with physical restraint (*r* = .31, *p* < .001) indicating that they were also more likely to be used alongside physical restraint.

**Table 3:** Frequency of conflict and aggression reported

	Incident Frequency
Aggression	227
Verbal	171*
Physical (person)	37
Physical (object)	19
Conflict	1340
Refusing regular meds	158
Refusing PRN	43
Demanding PRN	264
Smoking	27
Refusing to eat	138
Refusing to drink	70
Refusing to attend to personal hygiene	511
Refusing to get out of bed	53
Refusing to go to bed	42
Refusing to see worker	34
Containment	2233
Given PRN	178
Given IM	38
PICU transfer	20
Seclusion	237
Intermittent observation	1009
Constant observation	712
Staff show of force	11
Physical restraint	18*
Time-out	10

*Positive correlation with BWC use recorded

## Exploring the impact of BWCs utilising routinely collected ward data

### Acute ward results

Routine data collected via Datix records were used to examine differences in frequency of conflict and aggression, incident severity, and use of containment measures before, during, and after introduction of BWCs on each trial ward (see Table 4).

**Table 4:** Frequency of incidents across trial periods

Incident Type	Pre-trial		Trial		Post-trial	
	Acute Ward	PICU	Acute Ward	PICU	Acute Ward	PICU
Violence (against person)	19	23	19	16	20	36
Violence (against object)	7	3	6	4	3	3
Verbal aggression	6	1	3	0	6**	20**
Self-harm	4	3	1	6	7	0
Conflict	21	5	41	2	31	15

* *p* = .05

** *p* < .001

#### Incidents

There was no effect of trial period on incident type (*F*(10, 592) = 1.703, *p* = .077, Wilk’s Λ = 0.945), meaning there was no discernible difference in the type of incidents that occurred (E.g., verbal aggression, physical aggression) before, during, and after the pilot phase.

#### Incident outcomes

There was an effect of trial period on incident outcomes (*F*(6, 596) = 10.900, *p* < .001, Wilk’s Λ = 0.812). Incident severity was statistically significantly higher in the trial and post-trial periods compared to the pre-trial period. Use of restrictive practice was significantly lower in the post-trial period compared to the pre-trial and trial period. Police involvement was also lower in the post-trial period compared to the pre-trial and trial periods (see Table 5).

**Table 5:** Frequency of incident outcomes across trial periods

Incident Outcome	Pre-trial		Trial		Post-trial	
	Acute Ward	PICU	Acute Ward	PICU	Acute Ward	PICU
Severity (mean)	2.66	2.51	2.87*	2.47	2.84*	2.44
Use of restrictive practice	22	35**	35	11**	1**	9**
Police involvement	17	19	14	15	2**	4

* *p* = .05

** *p* < .001

### Results for the psychiatric intensive care unit

#### Incidents

There was an effect of trial period on incident type (*F*(10, 490) = 4.252, *p* < .001, Wilk’s Λ = 0.847). Verbal aggression was statistically significantly higher in the post-trial period compared to the pre and trial periods. Self-harm was statistically significantly higher in the trial period compared to the pre-trial and post-trial periods. There were no differences in violence against a person (*p* = .162), violence against an object or conflict behaviour (see Table 4).

#### Incident outcomes

There was a statistically significant difference in incident outcome across the trial periods (*F*(6, 494) = 12.907, *p* < .001, Wilk’s Λ = 0.747). There was no difference in incident severity or police involvement. However, use of restrictive practice was statistically significantly higher in the pre-trial period, reducing in the test period, and reducing further in the post-trial period (see Table 5).

### Qualitative findings

A total of 22 participants took part in interviews: five patients and 16 staff members. During the pre-pilot interviews a total of nine staff took part (five in the acute ward, four in the PICU ward) and two patients (both from the acute ward). After the pilot period, a total of eight staff took part (four from each ward) and three patients (all from the acute ward). Table 6 includes a full description of participants.

**Table 6:** Participants by timepoint and ward

	Time 1 (pre-pilot)		Time 2 (post pilot)			Total
	Acute Ward	PICU	Acute Ward		PICU	
Staff	5	4	4	4		17
Patients	2	0	3	0		5
Total per ward	7	4	7	4		22
Overall total	11		11			

Below we have presented the key themes aligning to the five core CFIR categories of Intervention Characteristics, Characteristics of Individuals, The Process of Implementation, the Inner Setting, and The Outer Setting (see Table 7).

**Table 7:** Overview of themes by CFIR Domain

CFIR Domain	Key themes
1.Intervention Characteristics	Design and usability of wearing a BWC on the ward
	Evidence Strength and Quality: do BWCs change anything?
	Relative advantage: are BWCs effective and efficient for the ward?
2.Characteristics of Individuals (Knowledge and Beliefs about the Intervention)	Staff and Patients knowledge and beliefs about the intervention.
	Perceived unintended consequences & impact on care
3.The Process of Implementation	Planning: Top-Down Implementation
	Execution: Training, Use and Ward Visibility
4.The Inner Setting	Ward Culture: Acceptance of Violence and Aggression is part of the job.
	Reactive nature of the ward and incidents
5.The Outer Setting	Resources: staffing
	Wider systemic issues

#### Intervention characteristics

##### Design and usability of wearing a BWC on the ward

When discussing the use of the BWCs, staff noted a range of design issues related to the cameras that they said impacted on their use and acceptance of the cameras. This included the nature of the camera pulling on clothing necklines (a particular issue for female staff working on male wards), and overheating causing discomfort and irritation to skin, challenges with infection control, as well as the issue of cameras in a mental health setting where they can be easily grabbed, thrown and broken during an incident. Staff often cited these design issues as related to the lack of proactive use of the cameras on the wards.

There were issues around the devices getting overheated or about it going on your clothing, it pulls down the top… we had one person who was leading on it, whenever he was around, of course, the camera was being used, but if he wasn’t there, people weren’t as proactive in using the camera. Petra (f), Staff, A, Post.

There were also issues with staff forgetting to wear the cameras, forgetting to switch them on during incidents, and forgetting to charge them at the end of the shift, reducing the potential use of the cameras by other staff. These were perceived as key logistical issues prior to the pilot and were reported as issues at the end of the pilot by several staff on the wards.

The practicalities of will they actually turn it on in those sorts of incidents, I don’t know. Just little stuff as well, like if they don’t put it back on the docking station, so you think you’re charging it for next shift but then it’s not charged and the battery is dead, that’s one less camera to use, so little stuff. Jamal (m), Staff, A, Pre.

In relation to usability, staff noted that the cameras were small and easy to use given their simple single switch interface. It was felt that not having to upload and manage the data themselves made cameras more user friendly and usable by staff members. Protocols put into place such as signing the cameras in and out, and allocation for use during shifts were likened to procedures in place for other security measures therefore the implementation of this for the BWCs was viewed as easy for many staff.

It’s just like the ASCOM alarms that we wear. There’s a system to sign in and sign out, and that’s it. Alice (f), Staff, A, Pre.

While staff were generally positive about the usability of the cameras, some were cautious of with concerns for those less confident with technology.

… you have to be conscious that there’s some people – it’s quite easy to use, but I can say that because I’m alright using devices and all that but there’s some that are older age or not that familiar with using devices that may struggle with using it… they’re feeling a bit anxious and a bit scared, if they’re not familiar with it then they won’t use it. Jamal (m), Staff, A, Pre.

##### Evidence strength and quality: do BWCs change anything?

There were conflicting reports regarding the potential benefits of using BWCs on the wards, with both staff and patients reporting mixed perceptions as to whether the cameras might reduce violence and aggression. In the pre-pilot interviews, some staff reported feeling that the BWCs may have a positive impact on reducing physical violence.

I think it’s going to reduce violence and aggression on the ward…I don’t think they’ll want to punch you…they might be verbally abusive but in terms of physical that might reduce. Sarah (f), Staff, I, Pre.

Patients however noted that the cameras might hold staff to account of their own behaviours and therefore may improve care, however they felt that this impact would wear off after the first few months after which people might forget about the cameras being there.

Now they’ve got the body cams, it’s going to be a lot of changes. They’ll think, ‘Ooh well he’s on tape’. So, it might do something to their conscience, they actually start to listen to patients… until the novelty wears off and it might go back to square one again. Ian (m), Patient, A, Pre.

One staff member suggested that incident rates had reduced following introduction of the BWCs, but they remained unsure as to whether this was due to the cameras, reflecting that violence and aggression on wards can be related to many factors.

I know our violence and aggression has reduced significantly since the start of the cameras pilot… I don’t know, because obviously wearing the camera’s one thing, but if they weren’t in use, I don’t know maybe just the presence of the camera made a difference. But yeah, it’s hard to tell. Petra (f), Staff, A, Post.

In contrast, several staff reported that they had seen limited evidence for such changes.

I used it yesterday. He was aggressive and I used it, but he even when I was using [it] he doesn’t care about the camera… it didn’t make any difference… It doesn’t stop them to do anything, this camera does not stop them to do anything. Abraham (m), Staff, I, Post.

Some staff suggested that in some circumstances the cameras increased patient agitation and created incidents, so there was a need to consider whether the BWCs were going to instigate aggression in some circumstances.

There has been with a few patients because they will threaten you. They will tell you, ‘if you turn it on, I’m gonna smash your head in’. So incidents like that, I will not turn it on… Yeah, or some of them will just tell you, ‘if you come close by, I’m going to pull that off your chest’. So things like that, I just stay back. Ada (f), Staff, A, Post.

One rationale for a potential lack of effectiveness was noted by both staff and patients and was related to the levels of acute illness being experienced by patients which meant that for many they were too unwell to have insight into their own actions or those of staff switching on the cameras.

We’ve had instances where patients are so unwell that they just don’t care. You switch on the camera, whether you switch it on or not, it doesn’t really change the behaviour. ‘All right, okay, whatever switch it on’. They’re so unwell, they’re not really understanding. Petra (f), Staff, A, Post.

It might make [staff] feel safer as a placebo effect, but I don’t think it would necessarily make them safer… I think the people that are likely to attack a member of staff are crazy enough that they’re not gonna even consider the camera as a factor. Harry (m), Patient, A, Pre.

This lack of evidence that the cameras were necessarily effective in reducing incident rates or severity of incidents may have had an impact on staff buy-in and the use of the cameras as a result. One staff member reflected that having feedback from senior management about the impact and evidence would have been useful during the pilot period to inform ward staff whether the cameras were influencing things or not.

Staff want feedback. I don’t think we’ve had any since we’ve had the cameras… it would be nice to get feedback from, I don’t know, whoever is watching it, and stuff like that. Ada (f), Staff, A, Post.

##### Relative advantage: are BWCs effective and efficient for the ward?

Due to a combination of personal beliefs related to BWCs, the lack of evidence of their impact on violence and aggression, and other elements of care and culture on the wards, a number of staff and patients explored alternative interventions and approaches that may be more beneficial than BWCs. Both staff and patients suggested that Closed Circuit Television (CCTV) as an intervention that provided the transparency of using cameras and video footage but with an independent perspective. This was felt by many to remove the bias that could be introduced in BWC use as the video capture didn’t require staff control of the filming.

I feel like [BWCs] puts all the power and trust into the hands of the staff and I feel that it would be better to have CCTV on the ward because CCTV is neutral. Harry (m), Patient, A, Pre.

I have control over that [BWC recording] … It kind of gives that split as well between staff and patients. You can tell me or I can tell you when to switch it on. Whereas I feel like a CCTV camera is there all the time. Nobody’s asking to switch it on. It’s there. If you wanted to review the footage you can request it, anyone can request to view the footage for a legitimate reason. Whereas the camera can come across as if you’re threatening. Petra (f), Staff, A, Post.

In addition, some participants reflected that the nature and design of BWCs meant that unless staff were present for an incident it wouldn’t be captured, whereas CCTV has the advantage of being always present.

If there’s CCTV, then it’s the same thing, you get me. Like, if its body worn cameras that people can always do things away from staff. They can always go down to that corridor to have their fight or go to the side where staff ain’t gonna see them to have their fight, but with CCTV you can’t do that. Elijah (m), Patient, A, Post.

In addition to exploring technological and video-based interventions, many staff noted that the key tool to violence reduction had to be the use of de-escalation skills, noting that the use of communication and positive relationships had to be the primary tool before other interventions such as BWCs or CCTV.

We do a lot of verbal de-escalation. So we got our destress room now still open. That has a punch bag, and it’s got sensory tiles, and the aim and hope is that when people do get frustrated, because we’re all human. We all get annoyed at anything or many little things in life. There is the aim that they go into that room and start punching the bag instead of property and damaging furniture. But we also are working really hard on verbal de-escalation and actually trying to listen to patients and talk to them before anything else. And that’s helped a lot. And between this kind of shared, or role modelling, where while we’re showing staff, actually even spending an extra 20 min is okay. If it means you’re not going to end up having to restrain a patient. Petra (f), Staff, A, Post.

By using communication skills and de-escalation techniques skilfully, some staff felt there was no need to utilise the BWCs. One concern with the introduction of the BWCs for staff was that the use of this technology may negatively impact on trust and relationships and the use of de-escalation.

Some situations I feel like it can make a situation worse sometimes… I think a lot of situations can be avoided if you just talk with people…. Trying to find out why they’re angry, trying to just kind of see it from their point of view, understand them… I think maybe additional training for verbal de-escalation is needed first. Patrick (m), Staff, A, Post.

#### Characteristics of individuals

##### Staff and patients’ knowledge and beliefs about the intervention

Overall, there were mixed views among both staff and patients as to whether cameras would reduce incidents, prior to and after the pilot period. When considering the possible impact on violence and aggressive incidents there was a view among staff that there was the need for a nuanced and person-centred view.

All the patients that come in, they’re different you know. They have different perceptions; they like different things… everyone is different. So, it just depends. We might go live, and then we have good feedback because the patients they are open and the understand why we have it, and then as they get discharged and new patients come in it might not go as well. It just depends. Serene (f), Staff, A, Pre.

As a result of the desire to be person-centred in the use of such interventions, one staff member noted that they weighed-up such consequences for the patient before using the BWC and would make decisions not to use the camera where they thought it may have a negative impact.

Actually, with this body worn camera, as I did mention, if a patient is unwell, that doesn’t, the patient will not have the capacity to I mean, say yes, you cannot just put it on like that. Yeah, I know it’s for evidence, but when something happens, you first have to attend to the patient. You first have to attend to the patient before this camera is, for me. Ruby (f), Staff, I, Post.

Some staff questioned the existing evidence and theories as to why BWCs work to reduce incidents, and instead noted that for some people it will instigate an incident, while others may be triggered by a camera.

I’m on the fence of how that is going to work because I know the evidence is that by telling a patient ‘look if you keep escalating I’m gonna have to turn this on’, but I know several of our patients would kind of take that as a dare and escalate just to spite so that you would turn it on. Diana (f), Staff, A, Pre.

In contrast, some staff felt the cameras helped them feel safer on wards due to transparency of footage as evidence for both staff and patients.

They [staff] need to use it for protection, for recording evidence, that type of thing… They can record instances for later evidence. Yeah, for them as well. Safer for them and for patients because you can also have the right to get them to record, because a patient might be in the wrong but sometimes it may be the staff is in the wrong position. And that’s achieving safety for patients as well. Yeah, I think it works both ways. Dylan (m), Patient, A, Post.

Positive buy-in was also related to the potential use of the intervention as a training, learning or reflective tool for staff to improve practice and care and promote positive staff behaviour.

If you know that your actions might be filmed one way or the other, that would make me to step up your behaviour to patients… if you know that your actions can be viewed, if the authority wants to, then you behave properly with patients so I think that will improve the quality of the care to patient. Davide (m), Staff, I, Pre.

While there were some positive attitudes towards the cameras, there remained considerable concerns among participants regarding the transparency of camera use to collate evidence in relation to incidents as it was widely noted that the cameras remain in staff control therefore there is an issue in relation to bias and power.

I do think my gut would say that it wouldn’t necessarily be well received. Because also I think people feel like prisoners in here, that’s how some of the patients have described their experience, so in terms of the power dynamic and also just – I think that can make one feel a bit, even worse, basically, you know? Leslie (m), Staff, A, Pre.

These issues lead to staff reporting they didn’t want to wear the camera.

I’d feel quite uncomfortable wearing one to be honest. Leslie (m), Staff, A, Pre.

The staff control of the cameras had a particular impact on patient acceptability of the intervention as it led to some patients viewing BWCs as being an intervention for staff advantage and staff safety, thus increasing a ‘them and us’ culture and leading to patient resistance to the cameras. This was particularly salient for those with prior negative experiences of police use of cameras or mistrust in staff.

I feel like the fact that the body worn cameras is gonna be similar to how the police use them, if a staff member has negative intent toward a patient, they would be able to instigate an incident and then turn the camera on and use the consequences of what they’ve instigated to expect restraint or injection or whatever else might happen. So, I feel like it would be putting all the power and trust into the hands of the staff and I feel that it would be better to have CCTV on the ward because CCTV is neutral. Whereas, the body worn camera, especially with some of the personality conflicts/bad attitudes, impressions I’ve had from certain members of staff since I’ve been here, I feel like body worn cameras might be abused in that way possible. Harry (m), Patient, A, Pre.

##### Perceived unintended consequences and impact on care

Prior to the implementation there were concerns from staff that the introduction of BWCs could have consequences beyond the intended use of reducing violence and aggression, unintentionally affecting a range of factors that may impact on the overall delivery of care. There was a key concern regarding the potential negative impact that cameras may have for patients who have paranoia or psychosis as well as for those who may have prior traumatic experiences of being filmed.

It might have negative impacts on these patients because I’m thinking about kind of patients with schizophrenia and things like that who already have paranoid delusions, thinking that people are after them, thinking that people are spying on them, people are watching them, and then seeing kind of cameras around. It might have negative impacts on them. Tayla (f), Staff, I, Pre.

When I was admitted I was going through psychosis… I don’t want to be filmed and things like that. So you just see a camera, a guy with a camera on, you are like, are you filming me? Elijah (m), Patient, A, Post.

There was also a considerable concern among both staff and patients that the use of cameras would have a negative impact on the therapeutic relationship between staff and patients. This was felt to be related to the implication that the cameras enhanced a ‘them and us’ dynamic due to the power differential that staff controlling the cameras can create, likened to policing and criminalisation of patients. With the potential of a negative impact on relationships between staff and patients, staff suggested they may be disinclined to use BWCs if it would stop patients speaking to them or approaching them if they needed support.

Yeah, I think it would probably damage [the therapeutic relationship] because I think what’s probably quite helpful is things that maybe create less of a power difference. I think to some extent, [the BWC] might hinder that ability. Like for example imagine going to a therapist and them just like ‘I’ve got this camera in the corner of the room and it’s gonna be filming our session and just in case – or like, just in case I feel that you might get aggressive with me’. Um, I don’t think that’s going to help the therapeutic relationship! Jamal (m), Staff, A, Pre.

When you get body worn cameras on there, the relationship as well between staff and patients, is just gonna instantly change because you’re looking like police! Elijah (m), Patient, A, Post.

In contrast, a minority of staff felt that the presence of cameras may improve relationships as they provide transparency of staff behaviour and would encourage staff to behave well and provide high quality care for patients.

It will also help how, improve the way we look at the patients… because if you know that your actions might be filmed one way or the other, that would make me to step up your behaviour you know… you behave properly with patients so I think that will improve the quality of the care to patient. More efficiently, more caring to patient. Davide (m), Staff, I, Pre.

#### The process of implementation

##### Planning: top-down implementation

Staff perceived that BWC implementation directives had been given by senior management or policy stakeholders whom they felt viewed the process from a position of limited understanding due to a lack of ‘frontline’ mental health service experience. This led to a lack of faith amongst staff, and a perception that funds were being misspent.

They sit up there, they just roll it out and see how it works, how it goes. They waste a whole lot of money, millions or whatever, thousands of pounds in it, and then they see that ‘Oh, it’s not gonna work’. They take it back and all of that. Before coming out with it, you need to come speak to us… they just sit up there drinking tea and coffee, and then they’re just like, Oh, yeah, well, let’s do it this way…come stay with these people, work with them, for just I give you a 12 h shift, stay with them. Richard (m), Staff, I, Post.

This was exacerbated when staff felt there was a lack of consultation or explanation.

we don’t always get the ins and outs of certain things…We know that the cameras are coming in and stuff like that, but you know, and obviously it’s gone through every avenue to make sure that it’s fine. But then sometimes we don’t always know the ins and outs to then explain to people why we have the cameras. Patrick (m), Staff, A, Post.

It was also highlighted that due to multiple initiatives being implemented and directives handed down in parallel, staff felt negative towards interventions more widely, with the BWCs being ‘*just another thing to do’*, adding to workload for staff and reducing enthusiasm to use the cameras.

it’s not just to do with the camera, I just think there’s lots of changes happening at once, and there’s loads of new things being constantly introduced that people are just thinking oh it’s another thing. I think that’s what it is more than the camera itself. Alice (f), Staff, A, Pre.

##### Execution: training, Use and Ward Visibility

Overall, there was a lack of consistency amongst staff in their understanding of the purpose and processes of using the BWCs on the wards.

What do you do, do you record every single thing or, I don’t know. Do you record like, if a patient said, I want to talk to you, confidential, you go sit in a room, do you record things like those or is it just violence and aggression? Ada (f), Staff, A, Post.

The lack of clarity regarding the purpose of the intervention and the appropriate use of the cameras was felt to impact staffs’ attitudes and acceptance of using them and contributed to a lack of transparency or perhaps trust regarding the use of any subsequent video footage.

I think if the importance of the recording was explained a bit more…and how it would improve things, I think people would use it more… that’s why I don’t think it’s always used sometimes… if you’re not sure why some of it’s important, then you’re not going to see the value…I think if you’re gonna keep with them, it’s about updating the training, teaching staff when to use it, then where does that information go? How does that look in terms of improving? Just a bit of transparency, I think. But when you don’t know certain things it’s a bit hard to get behind something or back it, you know? Patrick (m), Staff, A, Post.

The lack of information about the purpose and processes related to the intervention was also seen among patients, with most patients noting that they hadn’t received information about the cameras during their admissions.

No information at all. I don’t think any of the patients know about it. Toby (m), Patient, A, Post.

While training was provided it was widely felt that it was insufficient to provide understanding about the purpose of the cameras or the more in-depth processes beyond operational aspects such as charging and docking. Several staff interviewed were unaware of the training, while others noted that they had an informal run-through by colleagues rather than anything formal.

What training are you talking about?… I wasn’t here, so I was taught by my colleague. I mean, from what I was taught, to operate the camera, and to give a warning to the patient that you’re going to use the camera. Nevis (f), Staff, A, Post.

Longer training with further details beyond operational use was felt to be needed by staff.

I think the training should have to be longer, even if it’s like an hour or something… Like what situations deem the camera to be… more information on the cameras, when to use it, why it’s used, and I think if the importance of the recording was explained a bit more and what it was doing and how that recording would go and how it would improve things. Patrick (m), Staff, A, Post.

Furthermore, there was a need for training to be on a rolling basis given the use of bank staff who were not trained to use the cameras or to understand the proper processes or purpose of using the BWCs, which could leave them vulnerable to misuse or abuse.

We have bank staff [who aren’t trained] so they say ‘I don’t know how to use that camera you are giving me’. Nevis (f), Staff, A, Post.

#### The inner setting

##### Ward context: acceptance of violence and aggression is part of the job

It was widely believed by staff that the nature of working on a mental health ward included accepting that violence and aggression was part of the job. This was not seen as an acceptance of violence but more that the job was providing care for individuals who are mentally unwell, and confusion, fear, frustration and aggression can be part of that. As a result, there was an ambivalence among some staff that the introduction of cameras would change this.

I think like in this line of work, there’s always that potential for like risky behaviours to happen. I’m not sure if putting the camera on will make much difference. Patrick (m), Staff, A, Post.

Staff noted that because of the nature of the job, staff are used to managing these situations and they understood that it was part of the job; therefore, it was unlikely that they would record everything that on paper might be considered an incident.

There’s also enough things that happen here, so I don’t think they would record [the incidents] because it’s just another day here. You know what I’m saying… [staff] can just say, ‘Stop, go back to your room and leave it at that and that kind of be the end of it’. Dylan (m), Patient, A, Post.

We are trained for it. Eveline (f), Staff, I, Pre.

This acceptance that incidents are a hazard of mental healthcare was linked to staff’s acknowledgment that many factors make up the complexity of violence and aggression including the nature of individual patients, acuity levels, ward atmosphere, staffing levels, access to activities, leave and outside space. The interplay of multiple factors creates a context in which frustrations and incidents are likely, thus become part of the everyday and ‘normal’ life on the ward for staff and patients alike.

I feel like, you know, how in GP services you say, zero tolerance to abusive language, or any kind of harassment. I don’t think there is that on a psychiatric ward you are kind of expected to take all the abuse and just get on with it. Petra (f), Staff, A, Post.

With staff reported having a higher threshold for these behaviours it was perceived that this was likely to impact on the efficiency of the intervention as staff would be less likely to consider a situation as violent but more ‘*part of the job’*.

##### Reactive nature of the ward and incidents

Most participants noted that the ward context is always changing with people being admitted and discharged, with daily staff changes and wider turnover of staff, so things are never static and can change at any point. This reflects the dynamic nature of the ward which creates a complex moving picture that staff need to consider and react to.

[the atmosphere] it’s very good at the moment. If you had asked me this two weeks ago, I would say, ‘Oh, my gosh’. But it changes… The type of patient can make your whole ward change… it depends on the client group we have at the time. Nevis (f), Staff, A, Post.

Staff noted that a key limitation of using the cameras to reduce incidents was the reactive nature of the environment and care being provided. This was felt to impact on the feasibility and use of the cameras as staff noted that they often react to what is happening rather than thinking to ‘*put the camera on first*’. It was felt by staff with experience of reacting to incidents that the failure to use BWCs during these processes were linked to staff’s instincts and training to focus on patients as a priority.

Say for instance, you’re in the office, and two patients start fighting, or a patient attacks someone and, all you’re thinking about is to go there to stop the person. You’re not thinking about putting on any camera. You understand? So sometimes it’s halfway through it, somebody might say, ‘Has anybody switched the camera on’? And that’s the time you start recording… If something happens immediately, you’re not thinking about the camera at that time, you’re just thinking to just go, so yeah. Nevis (f), Staff, A, Post.

Incidents happen quickly and often surprise staff, therefore staff react instantly so are not thinking about new processes such as recording on the cameras as this would slow things down or is not in the reactive nature needed by staff during such incidents.

When you’re in the middle of an incident and your adrenaline’s high, you’re focusing on the incident itself. It’s very difficult for you to now remember, remind yourself to switch on the camera because you’re thinking, patient safety, staff safety, who’s coming to relieve you? What’s going on? Who’s at the door? Petra (f), Staff, A, Post.

In addition, the need for an immediate response meant that it was felt that by the time staff remember to, or have the chance to, switch the camera on it was often too late.

Sometimes in the heat of moments and stuff like that, or if the situation’s happening, sometimes you don’t always think to, you know, put your camera on. Patrick (m), Staff, A, Post.

#### Outer setting

##### Resources: staffing

Issues related to staffing were highlighted by several participants as a key problem facing mental health wards thus leading to staff having higher workloads, and higher rates of bank and agency staff being used on shift and feeling burnt-out.

Out of all the wards I’ve been on I’d say this is the worst. It’s primarily because the staff are overworked…it seems like they spend more time doing paperwork than they do interacting with the patients. Harry (m), Patient, A, Pre.

We’re in a bit of a crisis at the minute, we’re really, really understaffed. We’re struggling to cover shifts, so the staff are generally quite burnt out. We’ve had a number of people that have just left all at once, so that had an impact… Staff do get frustrated if they’re burnt out from lack of staff and what have you. Alice (f), Staff, A, Pre.

It was noted by one participant that the link of a new intervention with extra workload was likely to have a negative impact on its acceptability due to these increasing demands.

People automatically link the camera to then the additional paperwork that goes alongside it. It’s like, ‘Oh god, if we do this, we’ve got to do that’, and that could play a part. Petra (f), Staff, A, Post.

One staff member noted that the staffing issue meant there were more likely to be bank staff on wards so the care of patients may be affected as temporary staff may be less able to build meaningful therapeutic relationships.

So obviously there is the basic impact on safety of not having adequate staffing, but then there’s the impact of having a lot of bank staff. So obviously when you have permanent staff they get to know the patients more, we’re able to give them the more individualised care that we ideally should be giving them, but we can’t do that with bank staff. Diana (f), Staff, A, Pre.

It was also suggested that staffing levels and mix often made it more difficult to provide activities or facilitate escorted leave which can lead to patients feeling frustrated and becoming more aggressive.

So you know there is enough staff to facilitate the actual shift, so you know when there’s less staff like you say you’ve got people knocking at the door, but then you don’t have staff to take people out on leave straight away, that all has a rippling effect! Serene (f), Staff, A, Pre.

##### Wider systemic issues

Overall, there was a concern that the introduction of BWCs would not impact on wider, underlying factors that may contribute to frustration, aggression and incidents on wards. Providing a more enhanced level of care and better addressing the needs of patients was felt to be central to helping people but also reducing the frustration that patients feel when on the ward.

… for violence and aggression, [focus on] the mental health side of things like therapy and psychology should be compulsory. It shouldn’t be something you apply for and have to wait three or four weeks for. I think every person should, more than three or four weeks even, months even… we need psychology and therapists. That’s what will stop most violence, because psychologists and a therapist can edit the way that they speak to people because they’ve been given that skill depending on the way the person behaves. So that’s what we need regularly… not like all this dancing therapy, yoga therapy. That’s a person, that you come and you actually sit down and talk through your shit with them. That will help! Elijah (m), Patient, A, Post.

There’s a lack of routine and I think there’s a lack of positive interaction between the patient and the staff as well. The only time you interact with a member of staff is if you’re hassling them for something, you have to hassle for every little thing, and it becomes a sort of, frustration inducing and like I’m a very calm person, but I found myself getting very fucking angry, to be honest, on this ward just because out of pure frustration… there’s bigger problems than body worn cameras going on. Harry (m), Patient, A, Pre.

Staff agreed that there was a need to invest in staff and training rather than new technologies or innovations as it is staff and their skills behind the camera.

It’s not the camera that will do all of that. It’s not making the difference. It’s a very good, very beautiful device, probably doing its job in its own way. But it’s more about investing in the staff, giving them that training and making them reflect on every day-to-day shift. Richard (m), Staff, I, Post.

There was felt to be a need to support staff more in delivering care within wards that can be challenging and where patients are unwell to ensure that staff feel safe. While in some circumstances the cameras made some staff feel safer, greater support from management would be more beneficial in making staff feel valued.

## Discussion

In this study exploring the implementation and use of body-worn cameras on mental health wards, we employed two methods for collecting and comparing data on incidents and use of containment measures, including BWCs, on one acute ward and one psychiatric intensive care unit. We found no clear relationship between the use of BWCs and rates or severity of incidents on either ward. While BWCs may be used when there are incidents of both physical and verbal aggression, results indicate that they may also provoke verbal aggression, as was suggested during some interviews within this study. This should be a concern, as strong evidence that being repeatedly subject to verbal aggression and abuse can lead to burnout and withdrawal of care by staff [30]. These mixed findings reflect results that were reported in two earlier studies of BWCs on mental health wards [12, 13]. However, the very low use of the cameras, on just 10 per cent of the shifts where data was obtained, makes it even more difficult to draw any conclusions.

While the data shows limited impact of using BWCs on levels of incidents, we did find that during the pilot period BWC use tended to occur alongside physical restraint, but the direction of relationship is unclear as staff were asked to use BWCs when planning an intervention such as restraint. This relationship with restraint reflected the findings on several wards in a previous study [13], while contrasting with those reported in a second study that found reductions in incidents involving restraint during the evaluation period [12]. Such a mix of findings highlights the complexity of using BWCs as a violence reduction method within a busy healthcare setting in which several interacting components and contextual factors, and behaviours by staff and patients can affect outcomes [31]. The qualitative data collected during this pilot period highlighted the potential systemic and contextual factors such as low staffing that may have a confounding impact on the incident data presented in this simple form.

The findings presented within this evaluation provide some insights into the process of implementing BWCs as a safety intervention in mental health services and highlight some of the challenges and barriers faced. The use of implementation science to evaluate the piloting of BWCs on wards helps to demonstrate how multiple elements including a variety of contextual and systemic factors can have a considerable impact and thus change how a technology may vary not only between hospitals, but even across wards in the same hospital. By understanding the elements that may and do occur during the process of implementing such interventions, we can better understand if and how BWCs might be used in the future.

Within this pilot, extensive preparatory work conducted at a directorate and senior management level did not translate during the process of implementation at a ward level, which appeared to impact on the use of BWCs by individuals on the wards. This highlights that there is a need to utilise implementation science approaches in planning the implementation of new technologies or interventions and to investigate elements related to behavioural change and context rather than just the desired and actual effects of the intervention itself.

While ward staff and patients identified the potential for BWCs to enhance safety on the wards, participants distrusted their deployment and expressed concerns about ethical issues and possible harmful consequences of their use on therapeutic relationships, care provided and patient wellbeing. These themes reflect previous findings from a national interview study of patient and staff perspectives and experiences of BWCs in inpatient mental health wards [14]. Given these issues, alternatives such as increasing de-escalation skills were identified by staff as possible routes that may be more beneficial in these settings. Furthermore, other approaches such as safety huddles have also been highlighted within the literature as potential means to improve patient safety by looking ahead at what can be attended to or averted [32].

Furthermore, it is important to consider that the presence of power imbalances and the pre-existing culture on the ward have considerable implications for safety approaches and must be considered, as exemplified by the preferences by both staff and patients in this evaluation for more perceived ‘impartial’ interventions such as CCTV. As identified within previous studies [14], BWCs can have different implications for psychological safety, particularly for vulnerable patients who already feel criminalised in an environment with asymmetrical power imbalances between staff and patients. This is particularly salient when considering aspects of identity such as race, ethnicity, and gender both in terms of the identities of the patient group but also in terms of the staff/patient relationship.

While preferences in this study note CCTV as more ‘impartial’, work by Desai [33] draws on the literature about the use of surveillance cameras in other settings (such as public streets) as well as on psychiatric wards and concludes that CCTV monitoring is fraught with difficulties and challenges, and that ‘watching’ patients and staff through the lens of a camera can distort the reality of what is happening within a ward environment. In her recently published book, Desai [34] develops this theme to explore the impacts of being watched on both patients and staff through her ethnographic research in psychiatric intensive care units. She highlights concerns over the criminalisation of patient behaviour, safeguarding concerns in relation to the way women’s bodies and behaviours are viewed and judged, and the undermining by CCTV of ethical mental health practice by staff who attempt to engage in thoughtful, constructive, therapeutic interactions with patients in face-to-face encounters. Appenzeller et al.’s [35] review found that whilst the presence of CCTV appeared to increase subjective feelings of safety amongst patients and visitors, there was no objective evidence that video surveillance increases security, and that staff may develop an over-reliance on the technology.

In addition, our findings add to the existing literature which notes that alternative interventions and approaches that address underlying contextual and systemic issues related to improving care on inpatient wards require attention to address the underlying factors related to incidents, e.g., flashpoints [36]. Evidence suggests that factors leading to incidents can be predicted; therefore, there is a need to enable staff to work in a proactive way to anticipate and prevent incidents rather than view incidents as purely reactive [37–39]. Such skills-based and relational approaches are likely to impact more on improving safety and reducing incidents by addressing the complex and multi-faceted issue of incidents on inpatient mental health wards [40].

These findings highlight that interventions such as BWCs are not used within a vacuum, and that hospitals are complex contexts in which there are a range of unique populations, processes, and microsystems that are multi-faceted [41]. As a result, interventions will encounter both universal, specific, and local barriers that will impact on its functioning in the real world. This is salient because research suggests that camera use inside mental health wards is based on a perception of the violent nature of the mental health patient, a perception that not only influences practice but also impacts how patients experience the ward [33]. As a result, there needs to be careful consideration of the use of any new and innovative intervention aimed at improving safety within mental health settings that have limited research supporting their efficacy.

## Limitations

While the study provides important insights into the efficacy and acceptability of introducing BWCs onto inpatient mental health wards, there were several limitations. Firstly, the analysis of incident data is limited in its nature as it only presents surface level information about incidents without wider contextual information. Results using such data should be cautiously interpreted as they do not account for confounding factors, such as staffing, acuity, ward culture or ward atmosphere, that are likely to contribute to incidents of violence and aggression. For example, while there was a statistically significant decrease in restrictive practice on the PICU across the study period, we know that BWCs were not widely used on that ward, so this is likely due to a confounding variable that was not accounted for in the study design.

Secondly, the study faced limitations in relation to recruitment, particularly with patients. Researchers’ access to wards was challenging due to high staff turnover and high rates of acuity, meaning many patients were not deemed well enough to be able to consent to take part in the study. In addition, the low use of the cameras on wards meant that many patients, and some staff, had not seen the BWCs in use. Similarly, patients had been provided limited information about the pilot, so their ability to engage in the research and describe their own experiences with BWCs was restricted.

Thirdly, analysis captures the active use of the BWC, however it does not fully capture the impact of staff wearing the cameras even where they do not actively use them. While our qualitative analysis provides insight into the limitation of such passive use, it is likely that the presence of the cameras being worn by staff, even when turned off, may have an impact on both staff and patient behaviours. This may explain trends in the data that did not reach significance but warrant further investigation in relation to the presence of BWCs, nonetheless.

Finally, researchers had planned to collect quantitative surveys from staff and patients in relation to their experiences of the ward atmosphere and climate, views related to therapeutic relationships on the ward, levels of burnout among staff, views on care, and attitudes to containment measures. Due to issues related to staff time, patient acuity, and poor engagement from staff leading to challenges accessing the wards, the collection of such survey data was unfeasible, and this element of the study was discontinued. As a result, we have not reported this aspect in our paper. This limitation reflects the busy nature of inpatient mental health wards with pressures on staff and high levels of ill health among patients. As such, traditional methodologies for evaluation are unlikely to elicit data that is comprehensive and meaningful. Alternative approaches may need to be considered.

## Future directions

With BWCs being increasingly used across inpatient mental health services [14], it is important that further research and evaluation is conducted. To date, there is limited data regarding the effectiveness of this technology in relation to violence reduction; however, there may be other beneficial uses in relation to safeguarding and training [13]. Future research should consider alternative methods that ensure contextual factors are accounted for and that patient voices can be maximised. For example, focus groups with patients currently admitted to a mental health ward or interviews with those who have recently been on a ward that has used the cameras, would bypass problems encountered with capacity to consent in the present study. Furthermore, ethnographic approaches may provide a deeper understanding of the implementation, deployment and impact that BWCs have on wards.

## Conclusion

Overall, this research sheds light on the complexities of using BWCs as a tool for ‘maximising safety’ in mental health settings. The findings suggest that BWCs have a limited impact on levels of incidents on wards, something that is likely to be largely influenced by the process of implementation as well as a range of contextual factors, including the staff and patient populations on the wards. As a result, it is likely that while BWCs may see successes in one hospital site this is not guaranteed for another site as such factors will have a considerable impact on efficacy, acceptability, and feasibility. Furthermore, the findings point towards the need for more consideration to be placed on processes of implementation and the complex ethical discussions regarding BWC use from both a patient and a staff perspective.

In conclusion, while there have been advances in digital applications and immersive technologies showing promise of therapeutic benefits for patients and staff more widely, whether BWCs and other surveillance approaches are to be part of that picture remains to be seen and needs to be informed by high-quality, co-produced research that focuses on wider therapeutic aspects of mental healthcare.

### Electronic supplementary material

Below is the link to the electronic supplementary material.

Supplementary Material 1

Supplementary Material 2

Supplementary Material 3

Supplementary Material 4

Supplementary Material 5

Supplementary Material 6

## Data Availability

The data that support the findings of this study are available on request from the corresponding author. The data are not publicly available due to privacy or ethical restrictions.
