# Supplementary material for: Exploring the use of body worn cameras in acute mental health wards: a mixed-method evaluation of a pilot intervention
Source: BMC Health Serv Res. 2024 May 29;24:681. doi: 10.1186/s12913-024-11085-x (PMC11138092; doi:10.1186/s12913-024-11085-x)
Supplement: Supplementary file 1 — Supplementary Material 1 [file 12913_2024_11085_MOESM1_ESM.docx]

**Appendix B**

**Patient-Staff Conflict Checklist – Shift-Report – Revised (Bowers et al., 2003)**

| Date: |  | Ward: |  | Shift: |  |
| --- | --- | --- | --- | --- | --- |

| **Number of staff at start of shift** |  |
| --- | --- |
| Qualified: |  |
| Unqualified: |  |
| Bank/agency qualified: |  |
| Bank/agency unqualified: |  |
|  |  |
| **How many admissions were there during this shift?** |  |
| **How many incidents of aggression have there been during the shift?** |  |
| Verbal aggression |  |
| Physical aggression against objects |  |
| Physical aggression against others |  |
|  |  |
| **How many incidents of general rule-breaking have there been during the shift?** |  |
| Smoking in a no smoking area |  |
| Refusing to eat |  |
| Refusing to drink |  |
| Refusing to attend to personal hygiene |  |
| Refusing to get up and out of bed |  |
| Refusing to go to bed |  |
| Refusing to see workers |  |
|  |  |
| **How many incidents of drug or alcohol use have there been during the shift?** |  |
| Alcohol use (suspected or confirmed) |  |
| Other substance misuse (suspected or confirmed) |  |
|  |  |

| **How many incidents of absconding behaviour have there been during the shift?** |  |
| --- | --- |
| Attempting to abscond |  |
| Absconding (missing without permission) |  |
| Absconding (official report) |  |
|  |  |
| **How many incidents of self-harm have there been during the shift?** |  |
|  |  |
| **How many incidents of medication related behaviours have there been during the shift?** |  |
| Refused regular medication |  |
| Refused PRN medication |  |
| Demanding PRN medication |  |
|  |  |
| **How many incidents of self-harm have there been during the shift?** |  |
|  |  |
| **How many uses of these containment measures have there been during the shift?** |  |
| Given PRN medication (psychotropic) |  |
| Given IM medication (enforced) |  |
| Sent to PICU or ICA |  |
| Seclusion |  |
| Observation (intermittent) |  |
| Observation (constant) |  |
| Show of force |  |
| Physically restrained |  |
| Time out |  |
| Warning given that BWC will be switched on (but not used) |  |
| Warning given that BWCV will be switched on (used) |  |
| BWC switched on with no warning given |  |
