# Supplementary material for: Exploring the use of body worn cameras in acute mental health wards: a mixed-method evaluation of a pilot intervention
Source: BMC Health Serv Res. 2024 May 29;24:681. doi: 10.1186/s12913-024-11085-x (PMC11138092; doi:10.1186/s12913-024-11085-x)
Supplement: Supplementary file 3 — Supplementary Material 3 [file 12913_2024_11085_MOESM3_ESM.docx]

| **Staff Interview Schedule – at 6 month** |
| --- |
| **Introductory Questions** |
| 1. To start, could you tell me a bit about your job (e.g., what you do on a daily basis)?  Follow ups/prompts:   - Is your workload manageable? Why or why not? |
| 2. Could you tell me a bit about the ward? How are things at the moment?  Follow ups/prompts:   - Is it busy? What is staffing like? What is the atmosphere like? How much impact is COVID having now? |
|  |
| 3. Overall, what do you think relationships are like between staff and patients here?  *Follow ups/prompts:*   - Do you think patients are treated well here? Are they treated with respect and dignity? |
| 4. What do you know about body worn cameras?  Follow ups/prompts:   - What kind of information or training have you been given about body worn cameras, if any? |
| ***If the staff member has little or no knowledge about BWCs, explain:*** *BWCs are small, portable devices that staff can wear on their uniform to record incidents of violence and aggression while on the ward. Staff can choose when to turn the camera on or off, and service users can also request that the camera be turned on.* |
| 5. How do you think the use of body worn cameras has affected the ward atmosphere?   - How would you describe relationships between staff and service users? |
| **Implementation Questions** |
| 6. Who has been using the cameras on your ward? (E.g., bank staff, mental health nurses, HCAs, etc.) |
| 7. Have you used a body worn camera in your role? /Have you seen one used?  *Follow ups/prompts:*   - Can you tell me about the experience of using the camera? - How did it feel to use the camera? - Do you think it impacted behaviour? - What kind of debrief happens after a camera is activated? - How do you document that a camera has been used? |
| 8. How do you think patients are responding to the presence of body worn cameras? |
| 9. How are staff responding? (E.g., has there been any resistance?) |
| **Violence & Aggression** |
| 10. What kind of violence and aggression do you see on the ward (E.g., patient on staff, patient on patient, staff on patient)?  *Follow ups/prompts:*   - Do you know if all of these incidents get recorded as a formal incident? - How do you decide what incidents rise to the level of reportable? (E.g., severity of self-harm) - How do you decide when to record incidents? (What level makes it datix worthy) |
| 11. What violence and aggression reduction methods are used on the ward? (E.g., the Safewards model)  *Follow ups/prompts:*   - What kind of training have you received around these methods? - Are there any other things you think the ward needs to be doing to better address violence and aggression? |
| 12. How do you think body worn cameras might fit within those existing violence and aggression reduction methods? |
| 13. Do you think body worn cameras have reduced violence and aggression on the ward?  *Can you give me an example of this?* |
| **Safety** |
| 14. Do you think that body worn cameras have impacted staff safety, if so, how?  - can you give me some examples of this? |
| 15. How have body worn cameras impacted patient safety, if at all?  - can you give me some examples of this? |
| **Therapeutic Impact** |
| 16. What impact do you think body worn cameras have had on the therapeutic relationship between staff and patients, if any? |
| 17. Are there any situations or particular groups of patients that you think have been more affected by the use of body worn cameras in some way?  *Follow ups/prompts:*   - Informal patients or those detained under Mental Health Act? - Ethnicity, cultural background? - Those with a history of trauma? - In what way? |
| 18. Have the cameras been considered in care planning?  *Follow ups/prompts:*   - If so, how have they been used in care planning? - If not, why not? |
| 19. Have the body worn cameras been used to manage safeguarding concerns?  *Follow ups/prompts:*   - If so, how? - If not, why not? |
| **Logistical Questions** |
| 20. Can you use the camera everywhere on the ward?  *Follow ups/prompts:*   - Areas of the ward? Bedrooms? Bathrooms? - For managers: any logistical concerns about tracking use of cameras/footage? |
| 21. Do you have any concerns about the storage or use of footage from the body worn cameras?  *Follow ups/prompts:*   - Who gets to see the film? How secure it is? Used against staff?   22. How do you feel about the use of footage to prosecute patients? |
| 23. Are there any other aspects of life on the ward that you think have been impacted by the cameras?   - Workload? |
| 24. Is there anything else you would like to add about body worn camera use in mental health wards? |

Thank you very much.
