# Supplementary material for: Exploring the use of body worn cameras in acute mental health wards: a mixed-method evaluation of a pilot intervention
Source: BMC Health Serv Res. 2024 May 29;24:681. doi: 10.1186/s12913-024-11085-x (PMC11138092; doi:10.1186/s12913-024-11085-x)
Supplement: Supplementary file 4 — Supplementary Material 4 [file 12913_2024_11085_MOESM4_ESM.docx]

| **Service User Interview Schedule – at 6 months** |
| --- |
| **Introductory Questions** |
| 1. Would you mind telling me how long you have been on this ward and how has it been for you so far?  *Follow ups/prompts:*   - What has made it good/not so good for you? - Have you had previous admissions? Is it different this time? In what way? |
| 2. Can you tell me about the atmosphere on the ward currently, and how you think staff wearing body worn cameras may have impacted on your stay, if at all?  *Follow ups/prompts*   - Do you feel well-cared for? Why or why not? - Do you feel you have been treated with dignity and respect? |
|  |
| 3. Can you tell me what you know about body worn cameras?  *Follow ups/prompts:*   - What kind of information have you been given about body worn cameras, if any? - Do you feel that you understand why they are being used? - Have you experienced staff using body worn cameras? If so, can you tell me about that experience? - Do you feel that staff are well-trained and confident in using the cameras?   ***If the service user member has little or no knowledge about BWCs, explain:*** *BWCs are small, portable devices that staff can wear on their uniform to record incidents of violence and aggression while on the ward. Staff can choose when to turn the camera on or off, and service users can also request that the camera be turned on.* |
| **Implementation Questions** |
| 4. Have you seen the cameras being used on this ward?  *Follow ups/prompts:*   - If so, how often? - In what circumstances? - How did it feel when the staff member turned the camera on? - Do you think it impacted your behaviour? |
| - Have you ever asked for the camera to be turned on? Why?   *Follow up/prompts:*   - What was the outcome? - How did the staff member react? |
| 5. If you have been involved in a situation when a staff member turned the camera on, did you get to debrief with them afterwards?  *Follow ups/prompts:*   - If so, was it useful? - If not, would you have liked to? |
| **Violence & Aggression** |
| 6. Do you see any violence and aggression on the ward, if so, what kind (E.g., patient on staff, patient on patient, staff on patient)?  *Follow ups/prompts:*   - Verbal aggression, threats, damage to property, assaults? - Do you know if these incidents filmed on the cameras get recorded as a formal incident? |
| 7. Are you aware of staff using any methods or techniques to prevent or reduce violence and aggression on the ward?  *Follow ups/prompts:*   - Talking calmly to patients, going for a walk, de-escalation, medication, safety huddles, Safewards? - Are there any other things you think the ward needs to be doing to better address violence and aggression?   8. Have the cameras made things better, or worse? |
| - Changed how violence and aggression are prevented/managed? - How do you think body worn cameras fit within those existing violence and aggression reduction methods? |
| **Safety** |
| 9. Do staff wearing body worn cameras make you *feel* safe on the ward? If so, how? If not, why not?  - Do you think it's important that patient's behaviour is recorded by the camera? Can you give examples? |
| 10. Do you think body worn cameras make staff feel safer on the ward? If so, how? If not, why not? |
| **Therapeutic Impact** |
| 11. What impact have body worn cameras had on your relationships with staff, if any?  *Follow ups/prompts:*   - Do you think it has impacted how much you like staff? Why or why not? - Do you feel you can talk openly to staff wearing the cameras? Why/ why not? |
| 12. Are there any groups of patients or people with particular experiences you think have been differently affected by the use of body worn cameras?  *Follow ups/prompts:*   - *ethnicity, cultural background, religion, disability?* |
| **Logistical Questions** |
| 13. Are there any places you would like the cameras to be used/ time you wouldn’t want recorded?  *Follow ups/prompts:*   - *Areas of the ward? Bedrooms, bathrooms?* |
| 14. Are there any other aspects of life on the ward that have been impacted by the cameras? |
| 15. Do you have any concerns about the storage or use of footage from the body worn cameras?  *Follow ups/prompts:*   - Who gets to see the film? How secure it is?  1. How do you feel about the use of BWC footage to prosecute patients? 2. How do you feel about the use of BWC footage to be used against staff? |
| 16. Is there anything else you would like to add about the use of body worn cameras on mental health wards? |

Thank you very much.
