# Supplementary material for: Exploring the use of body worn cameras in acute mental health wards: a mixed-method evaluation of a pilot intervention
Source: BMC Health Serv Res. 2024 May 29;24:681. doi: 10.1186/s12913-024-11085-x (PMC11138092; doi:10.1186/s12913-024-11085-x)
Supplement: Supplementary file 5 — Supplementary Material 5 [file 12913_2024_11085_MOESM5_ESM.doc]

| **Service User Interview Schedule – Time 1** |
| --- |
| **Introductory Questions** |
| 1. Would you mind telling me how long you have been on this ward and how has it been for you so far?  Follow ups/prompts:   - What has made it good/not so good for you? - Have you had previous admissions? Is it different this time? In what way?   2. Can you tell me about the atmosphere on the ward currently?  *Follow ups/prompts*   - Do you feel safe on this ward? Why or why not? - Do you feel well-cared for? Why or why not? - Do you feel you have been treated with dignity and respect?   3. Do you feel like staff are safe on this ward? Why or why not?  4. Can you tell me what you know about body worn cameras?  *Follow ups/prompts:*   - What kind of information have you been given about body worn cameras, if any? - Have you experienced staff using body worn cameras? If so, can you tell me about that experience?   ***If the service user member has little or no knowledge about BWCs, explain:*** *BWCs are small, portable devices that staff can wear on their uniform to record incidents of violence and aggression while on the ward. Staff can choose when to turn the camera on or off, and service users can also request that the camera be turned on.* |
|  |
| **Violence & Aggression** |
| 5. What kind of violence and aggression do you see on the ward (E.g., patient on staff, patient on patient, staff on patient)?  *Follow ups/prompts:*   - Verbal aggression, threats, damage to property, assaults? - Do you know if these incidents get recorded as a formal incident?   6. Are you aware of staff using any methods or techniques to prevent or reduce violence and aggression on the ward?  *Follow ups/prompts:*   - Talking calmly to patients, going for a walk, de-escalation, medication, safety huddles, Safewards? - Are there any other things you think the ward needs to be doing to better address violence and aggression? |
| 7. Do you think body worn cameras might help at all in preventing or managing violence and aggression on the ward?  *Follow ups/prompts:*   - Might the use of cameras make things worse? - How do you think body worn cameras might fit within those existing violence and aggression reduction methods? |
| **Safety** |
| 8. What kind of impact do you think body worn cameras might have on your safety as a patient, if any?  *Follow up/prompts:*   - Would staff wearing body worn cameras make you *feel* safer? Do think they would make you safer on the ward? |
| 9. What kind of impact do you think body worn cameras might have on staff safety, if any?  *Follow ups/prompts:*   - Do you think they would make staff feel safer? Would they actually make them safer? |
| **Therapeutic Impact** |
| 10. What impact do you think body worn cameras might have on your therapeutic relationship with staff, if any?  *Follow ups/prompts:*   - Do you think it will impact how much you trust staff? Why or why not?   11. Are there any groups of patients or people with particular experiences you think might be differently affected by the use of body worn cameras?  *Follow ups/prompts:*   - *Sex, gender, ethnicity, cultural background, previous traumatic experiences?* |
| **Logistical Questions** |
| 12. Are there any limitations to using body worn cameras?  *Follow ups/prompts:*   - *Areas of the ward? Bedrooms, bathrooms?* |
| 13. Are there any other aspects of life on the ward that you think might be impacted by the cameras?  14. Do you have any concerns about the storage or use of footage from the body worn cameras?  *Follow ups/prompts:*   - Who gets to see the film? How secure it is? Used against staff? Used to prosecute patients? |
| 15. Are there any specific things you think we should be looking at in this research or anything else you would like to say?  Thank you very much. |
