# Supplementary material for: Exploring the use of body worn cameras in acute mental health wards: a mixed-method evaluation of a pilot intervention
Source: BMC Health Serv Res. 2024 May 29;24:681. doi: 10.1186/s12913-024-11085-x (PMC11138092; doi:10.1186/s12913-024-11085-x)
Supplement: Supplementary file 6 — Supplementary Material 6 [file 12913_2024_11085_MOESM6_ESM.doc]

| **Staff Interview Schedule – Time 1** |
| --- |
| **Introductory Questions** |
| 1. To start, could you tell me a bit about your job (e.g., what you do on a daily basis)?  *Follow ups/prompts:*   - How long have you worked here? - Is your workload manageable? Why or why not?   2. Could you tell me a bit about the ward? How are things at the moment?  *Follow ups/prompts:*   - Is it busy? What is staffing like? What is the atmosphere like? How much impact is COVID having now?   3. How safe is it on this ward?  *Follow ups/prompts:*   - Do you feel safe? Do you think the patients feel safe?   4. Overall, what do you think relationships are like between staff and patients here?  *Follow ups/prompts:*   - Do you think patients are treated well here? Are they treated with respect and dignity? |
| 5. What do you know about body worn cameras?  *Follow ups/prompts:*   - What kind of information have you been given about body worn cameras, if any? |
| ***If the staff member has little or no knowledge about BWCs, explain:*** *BWCs are small, portable devices that staff can wear on their uniform to record incidents of violence and aggression while on the ward. Staff can choose when to turn the camera on or off, and service users can also request that the camera be turned on.* |
| 6. How do you think the use of body worn cameras might affect the ward atmosphere?  *Follow ups/prompts:*   - Do you think it would impact on staff-patient relationships? - Do you think it would make staff or patients feel more or less safe? |
| 7. How do you think you would use body worn cameras in your role?  *Follow ups/prompts:*   - Would you be happy to wear and use a body worn camera? |
| **Violence & Aggression** |
| 8. What kind of aggression or violence do you see on the ward (E.g., patient on staff, patient on patient, staff on patient)?  *Follow ups/prompts:*   - Verbal aggression? Damage to property? Threats to patients or staff? Assaults? - Do you know if all incidents get recorded as a formal incident? - How do you decide what incidents rise to the level of reportable? (E.g., severity of self-harm, verbal aggression) |
| 9. What violence and aggression reduction methods are used on the ward?  *Follow ups/prompts:*   - E.g., de-escalation techniques, safety huddles, Safewards? - What kind of training have you received around these methods? - Are there any other things you think the ward needs to be doing to better address violence and aggression? |
| 10. How do you think body worn cameras might fit within those existing violence and aggression reduction methods?  *Follow ups/prompts:*   - Do you consider body worn cameras to be a containment measure or a form of restrictive practice? |
| 11. Do you think body worn cameras can reduce violence and aggression on the wards?  *Follow ups/prompts:*   - If so, can they do this alone, or in conjunction with other things? - If not, why not? |
| **Safety** |
| 12. What kind of impact do you think body worn cameras might have on staff safety, if any? |
| 13. What kind of impact do you think body worn cameras might have on patient safety, if any? |
| **Therapeutic Impact** |
| 14. What impact do you think body worn cameras might have on the therapeutic relationship between staff and patients, if any?  15. Are there any situations or particular groups of patients that you think might be more affected by the use of body worn cameras in some way?  *Follow ups/prompts:*   - Informal patients or those detained under Mental Health Act? - Sex, gender, ethnicity, cultural background? - Those with a history of trauma? - In what way? |
| 16. Do you think there is any potential benefit to using body worn cameras in care or safety planning?  *Follow ups/prompts:*   - If so, what are those benefits? - If not, why not? |
| 17. Do you think there is any potential benefit to using body worn cameras to manage safeguarding concerns?  *Follow ups/prompts:*   - Are there set ways to document safeguarding concerns? (E.g., if someone makes frequent complaints or accusations against staff, where does that information go? |
| **Logistical Questions** |
| 18. Are there any limitations to using body worn cameras?  *Follow ups/prompts:*   - Areas of the ward? Bedrooms? Bathrooms? - For managers: any thoughts about tracking use of cameras/footage? |
| 19. Do you have any concerns about the storage or use of footage from the body worn cameras?  *Follow ups/prompts:*   - Who gets to see the film? How secure it is? Used against staff? Used to prosecute patients? |
| 20. Are there any other aspects of life on the ward that you think might be impacted by the cameras? |
| 21. Are there any specific things you think we should be looking at in this research or anything else you would like to say? |

Thank you very much.
